# Newborn Hearing Screening in Bavaria—Is It Possible to Reach the Quality Parameters?

**DOI:** 10.3390/ijns4030026

**Published:** 2018-07-24

**Authors:** Inken Brockow, Kristina Söhl, Uta Nennstiel

**Affiliations:** Bavarian Health and Food Safety Authority (LGL), Veterinärstr.2, 85764 Munich-Oberschleißheim, Germany

**Keywords:** newborn hearing screening, tracking, screening center, congenital hearing disorder

## Abstract

Since the 1 January, 2009, newborn hearing screening (NHS) has been obligatory for every child in Germany. NHS is part of the Pediatrics Directive of the Federal Joint Committee. In this directive, details of the procedures and screening quality to be achieved are given. We evaluate if these quality criteria were met in Bavaria in 2016. The NHS data of children born in 2016 in Bavaria were evaluated for quality criteria, such as screening coverage in screening facilities, screening methods, referral rate (rate of failed tests at discharge) and a child’s age at the diagnosis of a hearing disorder. NHS was documented for 116,776 children born in Bavaria in 2016. In the first step, 78,904 newborns were screened with transient evoked otoacoustic emissions and 37,865 with automated auditory brainstem response. Of these, 9182 (7.8%) failed the first test in one or both ears. A second screening before discharge was performed on 53.3% of the newborns with a refer result in the first test, out of which 58.7% received a pass result. After the screening process, 4.6% of the newborns were discharged with a refer result. Only 18% of the first controls after discharge were performed by a pediatric audiologist. In 37.9% of the newborns, the screening center intervened to assure the control of any failed screening test. The median age of diagnosis for bilateral hearing loss was 5.3 months. In Bavaria, NHS was implemented successfully. A tracking system for all children who failed the hearing screening test is pivotal for early diagnosis and therapy of children with hearing deficiency.

## 1. Introduction

In Germany, the prevalence of a severe bilateral hearing disorder in infants is about 1–2 out of 1000 children [1,2]. If mild and unilateral hearing disorders are included, the prevalence is higher (2–3 per 1000) [2]. Early diagnosis and therapy improve speech, language and general development in children with bilateral hearing loss [3,4,5]. Therefore, a universal newborn hearing screening (NHS) was established in Germany by the Federal Joint Committee (“Gemeinsamer Bundesausschuss”, G-BA) in 2009 [6]. The aim of NHS is to identify newborns with a bilateral hearing disorder at a threshold level of 35 dB hearing level (HL) by the age of three months and to initiate therapy by the age of six months. Screening procedures and quality criteria are described in the Pediatrics Directive (“Kinder-Richtlinie”) by the Federal Joint Committee [7]. The defined aims of NHS are to screen more than 95% of all newborns. A two-step screening protocol is designed to achieve a less than 4% referral rate at discharge. Children with bilateral hearing loss should be diagnosed before the age of three months and therapy should be initiated at the age of six months.

In Bavaria, the screening center at the Bavarian Health and Food Safety Authority (LGL) is involved in the screening process to ensure that all newborns are screened (tracking for completeness) and that failed screening results are controlled (tracking for controls) [8]. Additionally, each year the screening quality is evaluated for the 125 maternity wards and 35 children’s hospitals in Bavaria. In this study, we evaluate if the quality parameters from the Pediatrics Directive could be reached in Bavaria in 2016.

## 2. Methods

### 2.1. Screening Protocol

In Germany, according to the Pediatrics Directive by the Federal Joint Committee, 95% of all newborns should be screened. A two-step screening protocol is established. The first step uses Transient Evoked Otoacoustic Emissions (TEOAE) or Automated Auditory Brainstem Response (AABR) testing for both ears. For children with risk factors for hearing impairment, screening with AABR is mandatory in the first step. Risk factors are defined in the German Pedaudiological Guideline following the definitions from the Joint Committee on Infant Hearing [9]. As a second step, 95% of the newborns that fail the first screening step in one or both ears should be screened using AABR testing before being discharged from hospital. Less than 4% of all screened infants should be discharged with a failed screening result from hospital (referral rate). A pediatric audiologist should perform a diagnostic test on these children before the age of 12 weeks and therapy should be initiated by the age of six months [7].

### 2.2. Population and Data Management

In Bavaria, NHS is part of one of the first obligatory medical check-ups for children and is therefore mandatory [10]. Data from all children that were born alive in Bavaria between 1 January and 31 December 2016, were used for this analysis. Data from all 125 maternity wards, 35 children’s hospitals, and some outpatient data are sent, mainly electronically, to the screening center of the Bavarian Health and Food Safety Authority (LGL) after parental consent was received. Data are stored in an ORACLE® database (Oracle-Version (12cR1) 12.1.0.2.0, ORACLE Deutschland B.V. & Co. KG, München, Germany). This data include personal information and information concerning the hearing screening tests, including the child’s age at testing, the test method, the test result and the screening facilities. Pediatricians, Ear Nose and Throat (ENT) specialist or pediatric audiologist sent the results from the follow-up testing after a failed screening by paper. A hearing disorder was defined according to the World Health Organization (WHO) as a permanent hearing threshold level greater than 25 dB HL [11]. Tracking for completeness was performed by the local health offices, in which the screening data of each child were compared with data from the birth register. The screening center tracks the necessary follow-up examinations after a failed screening or control testing result in one or both ears.

The main goal of this analysis was to evaluate if the aims of the Pediatrics Directive could be reached in Bavaria in 2016. Therefore, descriptive NHS parameters (rate of infants tested, method of testing according to the screening protocol, test results, infants discharged with failed screening (referral rate), follow-up testing, age at diagnosis and therapy) were extracted from the database and analyzed. Statistical calculations were performed using IBM SPSS Statistics for Windows, Version 23.0. (IBM Corp., Armonk, NY, USA) software. 

## 3. Results

A hearing screening was documented for 116,776 infants at the LGL screening center. This is a coverage rate of 98.7% of children living in Bavaria and born alive in 2016 according to the birth register. If all children born alive in Bavaria in 2016 were taken into account, including, for example, children living in Baden-Wurttemberg or Thuringia, the rate for NHS would be 94.9%. Fifty-one children were screened only after local health offices reminded the parents that a hearing screening was missing (tracking for completeness), and only 0.12% (129) of parents refused NHS for their child.

Infants were screened in the maternity ward, in the children’s hospital (113,663, 97.3%), or in outpatient settings (3113, 2.7%). Out of the 112,310 children for which the gestational age at birth was known, 1327 (1.2%) were born preterm, before 32 weeks of pregnancy. 85.7% of infants were screened during the first three days of their life, and 98.7% of preterm babies before the calculated date of birth. As a first step, a screening with TEOAE was performed on 78,904 children (67.6%) and with AABR on 37,865 children (32.4%). Out of all the children with a known risk factor for hearing disorders, 84.7% were screened with AABR. In the first step, 107,594 (92.1%) children passed the hearing screening. 53.3% of the 9182 children with a refer result in the first step of screening were re-tested before discharge from the hospital. This second step of screening was passed in 58.7% of cases. This second step of screening was performed in 51.7% of the tests with TEOAE and not with AABR. At the end of the screening process, 4.6% (5253) of infants were discharged with a refer result. The referral rate in the hospitals varied from 0.1% to 11.6%. In the outpatient settings, 5.5% (169) of children did not pass the hearing screening (refer result) and needed a control. Thirteen children needed follow-up examinations, because they first passed the screening but failed a later control testing.

After a refer result in the hospital, the first control test was performed on 24.7% of the children by a pediatrician, on 54.6% by an ENT specialist, and only on 19.2% by a pediatric audiologist. After this first control testing, only 21.2% of the children still had a remaining refer result in one or both ears. Up to twelve tests before diagnosis were documented at the screening center. In 37.9% of cases the screening center intervened to assure the control of a failed screening or control testing (tracking for controls). In 86% of children that failed the screening process, a hearing disorder was excluded. Only 8.0% of the children were lost to follow-up, and 0.5% couldn’t get a final diagnosis after they failed NHS because of other severe medical problems. In total, 121 (2.2%) children were diagnosed with a bilateral hearing disorder and 56 (1.0%) children with a unilateral hearing disorder. For 119 (2.2%) children the diagnostic process is still awaiting completion as of May 2018. The prevalence of a bilateral hearing disorder was 0.11%. The median age of diagnosis of a bilateral hearing disorder was 5.3 months of life (mean age 6.2 months, range 1–16).

## 4. Discussion

Most European countries have established a universal NHS program [12]. Many programs are organized according to the “1-3-6 plan” by the Joint Committee on Infant Hearing, consisting of the three goals: (1) Screening all infants no later than one month of age; (2) ensuring diagnostic audiological evaluation no later than three months of age for those who do not pass the screening; (3) enrolling infants identified with hearing loss in early intervention services no later than six months of age [13,14]. In Germany, universal NHS started in 2009, following the plan of screening in the first days of life, then three and six months of age for diagnosis and therapy, respectively. Further quality criteria were described in the Pediatrics Directive [7].

In Bavaria, universal NHS is well established. Since 2010, all Bavarian maternity wards and all children’s hospitals send the NHS data of screened children to the screening center at the LGL. A participation rate of 95% of the neonates is reached in Bavaria with a population-based coverage rate of 98.7%. Fifty-one newborns were screened only after the local health authorities reminded the parents that NHS was missing. A linkage of NHS data to either a birth register or the database for metabolic screening, which has a coverage rate close to 100% due to legal regulation, seems to be necessary to ensure that all neonates are screened, especially those who are not born in hospitals [15]. In Bavaria, tracking for completeness was especially important in the first years after the initiation of NHS in 2009. While 0.8% (746) of the children were only screened after the reminder of local health authorities in 2011 [16], in 2016 only 51 (0.04%) parents had to be reminded. Very high screening rates of up to 99.1% were achieved in some European regions and countries due to the structure of health care systems [17,18].

High rates of false-positive screenings result in unnecessary parental anxiety and subsequent follow-up diagnostics. Therefore, a referral rate of less than 4% is required according to the Pediatrics Directive. In Bavaria, the referral rate at discharge from the hospital was 4.6%. This is in accordance with other screening programs in Germany [19]. In Bavaria, there are still great differences in the referral rates between hospitals, as some wards reach referral rates of 0.1% while others have referral rates of 11.6%. The reasons for these differences are not well known, but hospitals with high referral rates normally do not perform a re-screening after a failed first screening. In addition, the staff turnover seems to be high on some wards. To reduce the rate of false-positive screening results the LGL gives feedback with a yearly quality report to every hospital and offers free training sessions.

In an effort to reduce parental anxiety and overall program costs, a two-step screening approach should be used [20]. With this approach, TEOAE screening is completed first, with an AABR done only for those newborns who do not pass the TEOAE screen. This protocol was recommended by the National Institute on Deafness and Other Communication Disorders Health Development Conference and published as Early Identification of Hearing Impairment in Infants and Young Children [21]. Large-scale studies have reported referral rates for two-step screening to be less than 2% [17,22,23]. It seems to be very important to follow this two-step screening process, as in our data the referral rate of 7.9% after the first screening could be reduced by a second screening to 4.6%. Unfortunately, only 53.5% of the Bavarian neonates received this re-screening before discharge and about 52% were tested with a second TEOAE screening and not the required AABR screening. One reason might be the increasingly early discharge from the maternity ward after an uncomplicated birth, leading to more failed hearing screening results on the first day of a neonate’s life and not allowing second-step screenings. Moreover, in Bavaria about 25% of maternity wards for healthy babies have no devices to screen with AABR. Furthermore, a screening with TEOAE is faster, easier to perform and less expensive than most AABR devices, as less expendable materials are needed. Therefore, it might be a reasonable discussion point to allow a second step screening with TEOAE after a failed first TEOAE in Germany, as it is already the screening protocol, for example, in the US and Great Britain [14,24]. The strict reinforcement of a two-step screening protocol [20], continuous checks on the quality of the screening and regular training sessions seem to be most effective to reduce false-positive screening results [18].

A high lost-to-follow-up rate acts as a threat to the overall success of NHS programs. The low lost-to-follow-up rate of 8% emphasizes the importance of a sufficient tracking system, such as the one established in Bavaria. In 37.9% of the newborns, an intervention by the screening center was conducted to assure the control of a failed screening or control test. Other countries or regions without a tracking system have to deal with lost-to-follow-up rates of up to 60% [25,26,27].

Referring to the Pediatrics Directive, a diagnostic follow-up after a failed hearing screening should take place before 12 weeks of age and should be performed by a pediatric audiologist. As there are very few pediatric audiologists in Bavaria, it is often an ENT specialist (54.6%) or a pediatrician (24.7%) that performs a first control or another screening. After this first control test, only 21.2% of the children still had a refer result in one or both ears (overall referral rate 1.7%). Many other screening programs and some other regions in Germany have this first control performed by an ENT or pediatric specialist to further reduce the number of children with false positive results [12,17,28]. Children that again fail this first control should be referred to a pediatric audiologist immediately to ensure an early diagnosis.

Until today, a bilateral hearing disorder (threshold level of >25 dB HL) was diagnosed in 121 children. For 119 children, the diagnostic process is yet to be completed, with the main reasons being fluid in the middle ear, other medical problems or parents that have to be reminded several times about the necessary confirmatory diagnostic. The prevalence of a bilateral hearing disorder was 1.1 of 1000 neonates, which is in a similar range to other studies [15,17,27,29]. Although the aim of NHS in Germany is primarily detecting bilateral hearing disorders at a threshold level of 35 dB HL, an additional 20 mild and 56 unilateral hearing disorders were discovered during the screening process. The median age of diagnosis of a bilateral hearing disorder was 5.3 months (mean age 6.2 months, range 1–16). In our study, age was not corrected for preterm babies, which might be a reason for a higher age than in other studies [18,28]. A diagnosis before the 12th week of life, as required by the Pediatrics Directive, was achieved only in 23.9% of children. Thus, it appears difficult to reach this quality criterion. Therapy was mostly initiated immediately after diagnosis. A late diagnosis occurred primarily in children with other medical problems, such as preterm children, children with syndromes or effusion in the middle ear.

## 5. Conclusions

A newborn hearing screening is documented for 98.7% of all Bavarian children born in 2016. The referral rate at discharge from hospital of 4.6% in 2016 could be further reduced by better reinforcement of the two-step screening protocol in all Bavarian hospitals. A first control test by ENT specialists or pediatricians before pediatric audiological diagnostics results in a drop in the referral rate to only 1.7%. A screening center, as it is established in Bavaria, is pivotal for a low loss-to-follow-up rate and an early diagnosis and therapy of children with hearing deficiency, since in 37.9% of the newborns the screening center had to intervene to assure the control of a failed screening test.
